# Raising genetic yield potential in high productive countries: Designing wheat ideotypes under climate change

**DOI:** 10.1016/j.agrformet.2019.02.025

**Published:** 2019-06-15

**Authors:** Nimai Senapati, Hamish E. Brown, Mikhail A. Semenov

**Affiliations:** a Department of Plant Sciences, Rothamsted Research, West Common, Harpenden, Hertfordshire, AL5 2JQ, United Kingdom; b The New Zealand Institute for Plant and Food Research, Private Bag 4704, Christchurch, New Zealand

**Keywords:** Climate change, Crop modelling, Ideotype design, Sirius model, Wheat, Genetic yield potential

## Abstract

Designing crop ideotype is an important step to raise genetic yield potential in a target environment. In the present study, we designed wheat ideotypes based on the state-of-the-art knowledge in crop physiology to increase genetic yield potential for the 2050-climate, as projected by the *HadGEM2* global climate model for the *RCP8.5* emission scenario, in two high-wheat-productive countries, *viz.* the United Kingdom (UK) and New Zealand (NZ). Wheat ideotypes were optimized to maximize yield potential for both water-limited (*IW_2050_*) and potential (*IP_2050_*) conditions by using Sirius model and exploring the full range of cultivar parameters. On average, a 43–51% greater yield potential over the present winter wheat *cv. Claire* was achieved for *IW_2050_* in the UK and NZ, whereas a 51–62% increase was obtained for *IP_2050_*. Yield benefits due to the potential condition over water-limitation were small in the UK, but 13% in NZ. The yield potentials of wheat were 16% (2.6 t ha^−1^) and 31% (5 t ha^−1^) greater in NZ than in the UK under 2050-climate in water-limited and potential conditions respectively. Modelling predicts the possibility of substantial increase in genetic yield potential of winter wheat under climate change in high productive countries. Wheat ideotypes optimized for future climate could provide plant scientists and breeders with a road map for selection of the target traits and their optimal combinations for wheat improvement and genetic adaptation to raise the yield potential.

## Highlights

• Wheat ideotypes were designed to increase genetic yield potential in 2050-climate.
• Ideotypes were optimized for two high productive countries *viz*. UK and New Zealand.
• A 43–62% greater genetic yield potential was achieved for wheat ideotypes.
• Yield potentials were 16–31% greater in New Zealand than in the UK.
• Ideotype designs provide a road map for raising genetic yield potential in wheat.

## Graphical abstract

Study sites and simulated grain yield of local winter wheat *cv*. *Claire* in the baseline- (*CL_Base_*) and 2050-climate (*HadGEM2*, *RCP8.5*) (*CL_2050_*), and wheat ideotypes designed for raising genetic yield potentials under 2050-climate in water-limited (*IW_2050_*) and potential (*IP_2050_*) conditions. The box plots show the 5-, 25-, 50-, 75- and 95-percentiles including mean. UK: ED - Edinburgh, LE- Leeds, RR-Rothamsted; NZ: GO-Gore, LI-Lincoln, PU-Pukekohe.

**Figure:**
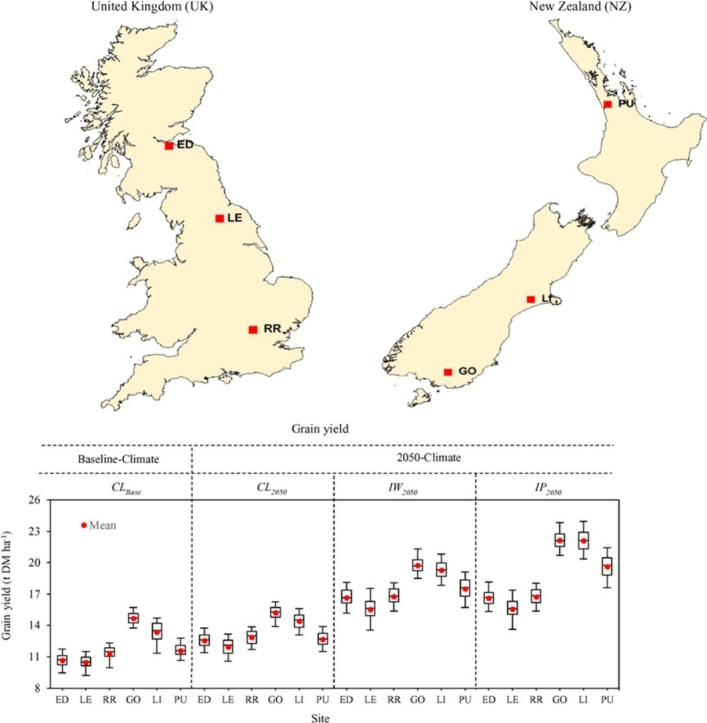

## 1 Introduction

To ensure food security for the world’s growing population, food production will need to increase by around 70% by 2050 (FAO, 2009, 2014). Wheat (*Triticum aestivum* L.) is one of the key staple crops in global food security, providing about 20% of total dietary calories and protein needs, with about 730 million tonnes of annual production from a harvested area of around 2.1 million km^2^ globally (FAO, 2016; Shiferaw et al., 2013). A trend of stagnating yields has already been observed around the world for staple crops, including wheat (Brisson et al., 2010). The widespread degradation of land and the exhaustion of water and other natural resources are challenging the sustainability of current food production systems (FAO, 2014). At the same time, the combination of ongoing climate change, including increasing air temperature, changing precipitation patterns and quantities, the increasing frequency and severity of extreme climatic events and adverse weather conditions, threatens present food production and any future targets (Asseng et al., 2015; Trnka et al., 2015; Zampieri et al., 2017). With the limited scope for extending present crop-growing areas, a considerable increase in crop productivity is required to guarantee future food security in the face of ongoing climate change (Reynolds et al., 2011). Increasing the upper limit of genetic yield potential is one of the key components of an integrated approach to improve crop productivity, besides optimization of agronomic management and sustainable intensification (Godfray et al., 2010, Reynolds et al., 2011, 2009).

Crop yield is a quantitative trait controlled by many plant traits, where most of the traits are polygenic in nature (Shi et al., 2009; Wu et al., 2012). Raising the upper limit of genetic yield potential through traditional plant breeding has remained very successful, for example, increasing wheat yields by using gene encoding for the dwarfing of plants in the Green Revolution (Hawkesford et al., 2013; Reynolds et al., 2009). However, the rate of success of the traditional plant breeding, which is generally defined as “selection for yield”, is heavily dependent on the availability of a wide range of parents, the choice of the crosses to be made and the skilful evaluation of the emergent genotypes, together with one’s share of good fortune (Donald, 1968). Conventional breeding is constrained by time and resources and is, thus, less efficient in terms of progress achieved, as desirable traits come mostly by chance without the underlying physiology being fully understood (Fischer, 2007). Breeding for new cultivars of high yield potential in a target environment, such as future climate change, is a different and challenging task for plant breeders as a) understanding the physiological basis of yield potential in a changing environment is required, b) selection of desirable traits and their exact combinations for future improvements is difficult, and c) reproducing future climatic conditions for evaluation of the performance of a new cultivar is difficult. Designing crop ideotypes based on the state-of-the-art knowledge in crop physiology and assessment of their performances in the target environment beforehand could help in breeding crops for high yield potential under climate change. Donald (1968) first proposed the idea of ‘breeding of crop ideotypes’, in which breeders select plant ideotypes based on their knowledge of crop physiology for improvement of plant traits in the target environment, and then breed for them. A crop ideotype is a virtual idealized crop, or a crop model, that is expected to produce a greater quality and quantity of grain yield when developed as a cultivar. Designing crop ideotypes *in silico* has gradually become a reality with the substantial increase in computational power of modern computers and the significant improvements in process-based eco-physiological crop models (Donald, 1968; Senapati et al., 2018; Stratonovitch and Semenov, 2015; Tao et al., 2017). Crop modelling is the most powerful tool for designing such crop ideotypes in target environments. Crop models (a) are efficient in designing crop ideotypes in terms of time and resources, (b) help in selecting optimal combinations of target traits when considering possible trade-offs between them, (c) assess performance of potential candidates across target environments, (d) assist in deconvoluting complex traits, such as crop yield, to a list of simpler component traits suitable for further analysis and improvement (Gouache et al., 2017; Hammer et al., 2005; Martre et al., 2015; Rötter et al., 2015; Tao et al., 2017). In the present study, we designed wheat ideotypes for raising genetic yield potential under future climate using Sirius, a process-based wheat model coupled with a powerful computational framework for ideotype optimization (Jamieson et al., 1998b; Semenov and Stratonovitch, 2013; Stratonovitch and Semenov, 2010). Sirius was extensively calibrated and validated for many modern wheat cultivars, and it performed well under diverse climatic conditions across Europe, the USA, Australia and New Zealand, including Free-Air CO_2_ Enrichment experiments (Asseng et al., 2015; Jamieson et al., 2000; Jamieson and Semenov, 2000; Lawless et al., 2005; Martre et al., 2006; Semenov et al., 2007, 2009; Stratonovitch and Semenov, 2010).

High-wheat-productive countries are situated at high latitudes for various reasons, such as cooler seasonal temperature, sufficient rainfall, long crop-growing season, high cumulative intercepted radiation, etc. The United Kingdom (UK) and New Zealand (NZ) are two of the high productive countries at high latitude, with an average national wheat yields of ≥8 t ha^−1^ over the last decade (Defra, 2017; FAOSTAT, 2018). These two countries have been competing for the Guinness World Records for wheat yield (16–17 t ha^-1^) for the last decade (GWR, 2015, 2017). However, these countries are characterized with opposite wheat growth calendar because of their positions in different hemispheres with different latitudes, *viz*. northern hemisphere (UK) and southern hemisphere (NZ). Thus, these two countries together provide a unique opportunity to investigate the possibility of raising the upper threshold of wheat yield in contrasting growing conditions.

Although there are some studies on the quantitative design of ideotypes for high yield potential of winter cereals under current or future climatic conditions, either they involve local or manual optimization of only a few traits (e.g., wheat ideotypes by Sylvester-Bradley et al., 2012), or they do not explore the full parameter space for optimization (e.g., barley ideotypes by Tao et al., 2017). Ideotypes for a target environment need to be optimized in a global multidimensional cultivar parameter space exploring in full the parameter ranges and their possible interactions. Although a few studies have assessed the quantitative yield potentials of wheat ideotypes under future climatic conditions in Europe, the ideotype design was not focused on shifting the upper threshold of genetic yield potential, particularly in high wheat productive countries at high latitude (Semenov et al., 2009; Semenov and Shewry, 2011; Semenov and Stratonovitch, 2013; Senapati et al., 2018; Stratonovitch and Semenov, 2015). The objective of this study was to design wheat ideotypes to increase the genetic yield potential in the 2050-climate as projected by the *HadGEM2* global climate model (GCM) for the *RCP8.5* emission scenario in two high-wheat-productive countries, *viz*. the UK and NZ. The design methodology was based on the knowledge of crop physiology, the Sirius wheat simulation model and evolutionary optimization in the multidimensional cultivar parameter space.

## 2 Material and methods

### 2.1 Target sites

**Fig. 1:**
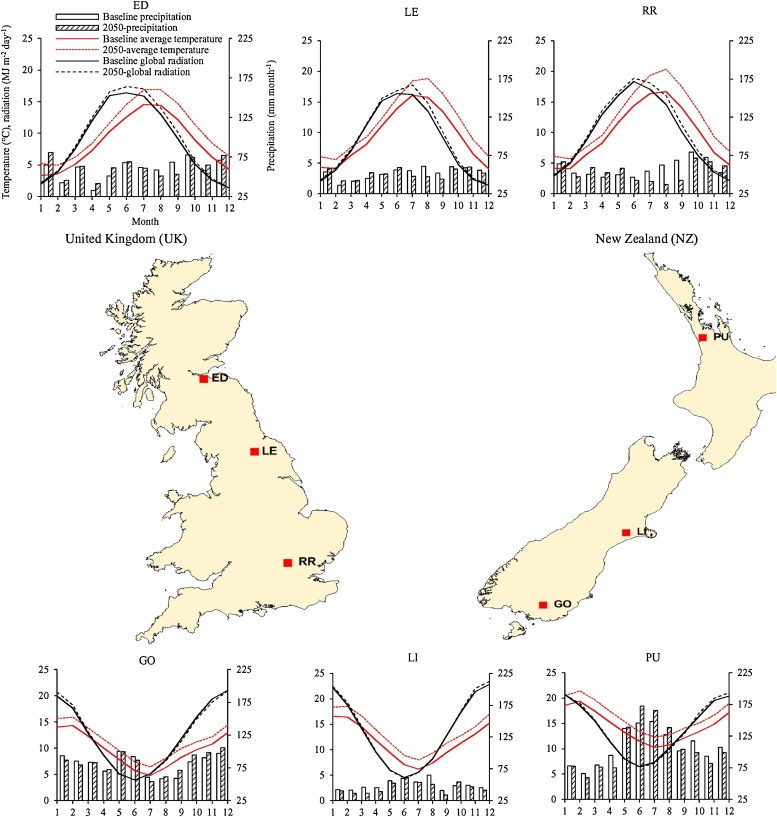
Location of study sites across the United Kingdom (UK) and New Zealand (NZ). ED: Edinburgh (UK), LE: Leeds (UK), RR: Rothamsted (UK), GO: Gore (NZ), LI: Lincoln (NZ), PU: Pukekohe (NZ). The baseline-climate, and the 2050-climate (*HadGEM2*, *RCP8.5*) *viz*. mean air temperature, mean monthly precipitation and mean global radiation. Note that the UK (northern hemisphere) and NZ (southern hemisphere) are in opposite hemispheres.

For the present study, two high wheat-productive countries, with mean national yields of ≥8 t ha^−1^ (FAOSTAT, 2018), were selected from two different hemispheres, *viz*. the UK in the northern hemisphere and NZ in the southern hemisphere. Three sites were selected across major wheat-growing regions in the UK, covering high (northern) (Edinburgh: ED) and medium (Leeds: LE) to low (southern) (Rothamsted: RR) latitudes. Similarly, three sites were selected across major wheat-growing regions in NZ, covering high (southern) (Gore: GO) and medium (Lincoln: LI) to low (northern) (Pukekohe: PU) latitudes (Fig. 1). Table 1 shows the detailed site characteristics.

**Table 1:** Characteristics of the study sites representing major wheat-growing regions across the United Kingdom (UK) and New Zealand (NZ).

ID	Site	Country^†^	Latitude	Longitude	Air temperature^††^	Precipitation^††^	Global radiation^††^
			(°)	(°)	(°C)	(mm yr^−1^)	(MJ m^−2^ day^-1^)
ED	Edinburgh	UK	55.94	−3.31	8.6	717	8.7
LE	Leeds	UK	54.30	−1.53	9.5	626	8.6
RR	Rothamsted	UK	51.80	−0.35	9.8	700	9.8
GO	Gore	NZ	−46.12	168.89	9.8	976	12.4
LI	Lincoln	NZ	−43.70	172.00	11.6	596	13.6
PU	Pukekohe	NZ	−37.21	174.86	14.5	1296	14.0

^†^Note that the UK (United Kingdom) and NZ (New Zealand) are in the northern hemisphere and southern hemisphere respectively.

^††^Mean baseline climatic condition for 1980–2010.

### 2.2 Baseline and target 2050-climate

30-years (1980–2010) of daily observed weather data at each study site were used for estimating site parameters for the baseline climate. To assess inter-annual variation in crop production in the baseline climate, a stochastic weather generator (*LARS-WG 6.0*) (Semenov and Stratonovitch, 2010, 2015) was used to generate 100 years of daily weather data at each site using the baseline site parameters, hereafter defined as ‘baseline-climate’, or the ‘current-climate’, with an atmospheric CO_2_ concentration of 364 ppm. The ability of the *LARS-WG* weather generator to reproduce climatic variability and climatic extreme events was thoroughly tested in diverse climates (Semenov, 2007, 2008; Semenov et al., 1998). The baseline-climate at each site was used for evaluation of the performance of a local winter wheat cultivar in present conditions. The target 2050 climate at each site was based on projection from the *HadGEM2* global climate model from the CMIP5 ensemble (Taylor et al., 2012) for the period 2040–2060 for the Representative Concentration Pathway of 8.5 (*RCP8.5*), with an atmospheric CO_2_ concentration of 541 ppm (Riahi et al., 2011). Similar to the baseline, 100 years of daily weather data for 2050 were generated at each site by using *LARS-WG 6.0*, hereafter defined as the ‘2050-climate’ (*HadGEM2*, *RCP8.5*). The daily 2050-climate data were used as target climatic conditions to design wheat ideotypes and evaluate their performances in comparison to the baseline.

In the baseline-climate, mean annual air temperature, annual precipitation and mean daily global radiation were 9.3 °C, 681 mm yr^−1^ and 9.0 MJ m^-2^ day^−1^ respectively in the UK, and 12 °C, 956 mm yr^−1^ and 13.3 MJ m^-2^ day^-1^ respectively in NZ (Fig. 1, Table 1). This represents 2.7 °C, 40% and 48% greater mean annual air temperature, mean annual precipitation and daily mean global radiation, respectively in NZ compared to the UK. There were increasing trends in temperature and global radiation between sites from high (northern) to low (southern) latitudes in the UK (ED–RR). Similar trends were also found among sites in NZ from high (southern) to low (northern) latitudes (GO–PU). In the UK, annual precipitation was almost equally high at ED and RR, and lowest at LE, whereas annual precipitation for NZ was highest at PU and lowest at LI. In the 2050-climate, averaged air temperature and global radiation increased compared to the baseline-climate by 2.1 °C and 6.7% respectively in the UK, and 1.9 °C and 1.1% respectively in NZ, whereas the decrease in annual precipitation was small (<2%) in both the UK and NZ (Fig. 1). In the 2050-climate scenario, averaged air temperature, precipitation and global radiation are 2.4 °C, 41% and 40% greater in NZ than in the UK.

### 2.3 Sirius model

Sirius is a process-based wheat simulation model with a daily timescale. It includes an optimization framework, which facilitates designing ideotypes and optimization of cultivar parameters and plant traits for target environments (Senapati et al., 2018). Sirius utilizes the multicore architecture of modern computers, which substantially reduces the running time for computationally intensive applications such as ideotype design. The model requires daily weather data, a cultivar description, a soil physical description and management information as model inputs. Sirius consists of various sub-models that describe soil, plant phenological development, water and nitrogen (N) uptake, photosynthesis, and biomass and grain production. The model also includes the effects of abiotic stress (*e.g*., common heat and water stresses), N limitation, and drought and heat stress during reproductive development on photosynthesis, biomass production and grain yield. Table 2 shows the important cultivar parameters used in Sirius. Briefly, photosynthesis and biomass production are simulated on a daily basis as the product of intercepted photosynthetically active radiation (*PAR*) and radiation use efficiency (*RUE*), limited by temperature and water stress. Phyllochron (*P_h_*) and day length response (*P_p_*) control the rate of crop development. Phenological development is calculated from the mainstem leaf appearance rate and final leaf numbers, with the latter determined by responses to day length and vernalisation. Canopy is described as a series of leaf layers associated with individual mainstem leaves. Leaf area development in each layer is simulated by a thermal time sub-model, and actual leaf area is calculated using a simple limitation rule. Leaf senescence is expressed in thermal time and linked to the rank of the leaf in the canopy. Total canopy senescence synchronizes with the end of grain filling. Leaf senescence could be accelerated by N limitation to sustain green leaves and grain filling, or by abiotic stress, *viz*. temperature or water stresses. Soil is described as a cascade of 5-cm layers up to a user-defined depth. Photosynthesis and new biomass production are reduced by water stress proportionally to the response of photosynthesis to water stress. The water stress reduces photosynthesis and biomass production during the crop-growing period. Water stress also increases the rate of leaf senescence, which ultimately reduces the grain yield. Drought and heat stresses, especially during the reproductive phase, decrease the primary fertile grain setting number as a result of the abnormal development of both ovary and anthers, the premature abortion of florets, and the irreversible abortion and sterility of both male and female reproductive organs and gametophytes. Sirius computes a drought stress factor (*DSF*) and heat stress index (*HSI*) to estimate the effects of drought and heat stresses respectively on the primary grain setting number during reproductive development. Additionally, the potential weight of grain could be limited by heat stress and water limitation during grain filling and endosperm development. A detailed description of the Sirius model can be found elsewhere (Brooks et al., 2001; Jamieson and Semenov, 2000; Jamieson et al., 1998b; Lawless et al., 2005; Semenov and Stratonovitch, 2013; Senapati et al., 2018; Stratonovitch and Semenov, 2015).

**Table 2:** Description of the Sirius calibrated parameters of the local winter wheat *cv*. *Claire.*

No.	Parameters	Symbol	Unit	Value
1	Phyllochron	*P_h_*	ºC day	110.0
2	Day length response	*P_p_*	Leaf h^−1^day length	0.5000
3	Thermal time from sowing to emergence	*TT_SOWEM_*	ºC day	150.0
4	Thermal time from anthesis to beginning of grain fill	*TT_ANBGF_*	ºC day	100.0
5	Thermal time from beginning of grain fill to end of grain fill	*TT_BGFEGF_*	ºC day	650.0
6	Thermal time from end of grain fill to harvest maturity	*TT_EGFMAT_*	ºC day	200.0
7	Maximum area of flag leaf	*A_Max_*	m^2^ leaf m^−2^ soil	0.0070
8	Minimum possible leaf number	*L_Min_*	–	8.0
9	Absolute maximum leaf number	*L_Max_*	–	18.0
10	Response of vernalisation rate to temperature	*VAI*	Day^−1^°C^−1^	0.0012
11	Vernalisation rate at 0 °C	*VBEE*	Day^−1^	0.012
12	Heat stress grain number reduction threshold temperature	*HSGNT*	ºC	30.0
13	Heat stress grain number reduction rate	*HSGNR*	ºC^−1^	0.04
14	Drought stress grain number reduction stress threshold	*DSGNT*	–	0.90
15	Drought stress grain number reduction stress saturation	*DSGNS*	–	0.30
16	Maximum drought stress grain number reduction	*DSGNRMax*	–	0.20
17	Maximum potential grain weight	*MaxGW*	g	0.045
18	Grain number per g DM ear	*GNEar*	g^−1^	100
19	Stay green	*S_G_*	–	0.50
20	Rate of root water uptake from the root bottom	*R_u_*	%	3.0
21	Response of photosynthesis to water stress	*W_sa_*	–	0.500
22	Maximum acceleration of leaf senescence due to water stress	*W_ss_*	–	1.270

### 2.4 Designing wheat ideotypes for raising genetic yield potential for the 2050-climate

In the present study, a crop ideotype was defined as a subset of Sirius cultivar parameters that would deliver high yield performance in a target environment when optimized (Table 3). A cultivar, which is based on an ideotype and utilizes its optimal combination of trait values, would deliver optimal yields for the environment in question. *Claire* is a popular winter wheat variety in Europe, including the UK, and in NZ for its soft milling, early sowing, lodging and disease-resistant characteristics, and consistent high yield performance (Limagrain, 2018; Powell et al., 2013). For the same reasons, *Claire* has been used extensively as a parent in many wheat breeding programmes (Powell et al., 2013). In the present study, the local winter wheat *cv*. *Claire* was used as a parent for designing wheat ideotypes. The Sirius model was calibrated for *Claire* as being heat- and drought-sensitive during reproductive development. A list of the 22 Sirius cultivar parameters as calibrated for *Claire* is presented in Table 2. In our study, only eight cultivar parameters were selected from the above list for optimization (Table 3). As winter wheat is mostly grown under rainfed condition in the UK and NZ, we designed a wheat ideotype for the water-limited condition (rainfed) under 2050-climate (*IW_2050_*). Additionally, as irrigation is also available in NZ at present and will probably be available in the future, we designed a wheat ideotype for the potential condition (irrigated or no-water limitation) under 2050-climate (*IP_2050_*). Both the ideotypes, *viz*. *IW_2050_* and *IP_2050_*, were optimized to raise the genetic yield potential under 2050-climate in the UK and NZ.

**Table 3:** Sirius cultivar parameters used for designing wheat ideotypes for raising genetic yield potentials under the 2050-climate, and genetic variations observed in those parameters for wheat.

Parameters	Symbol	Unit	Range used in model optimization	Genetic variation	Reference
***Phenology***					
Phyllochron	*P_h_*	ºC day	70–120	≤20%	Ishag et al. (1998); Mosaad et al. (1995)
Day length response	*P_p_*	Leaf h^−1^day length	0.065–0.900	9.74–107.40*	Kosner and Zurkova (1996)
Duration of grain filling	*G_f_*	ºC day	500–900	≤40%	Akkaya et al. (2006); Charmet et al. (2005); Robert et al. (2001)
***Canopy***					
Maximum area of flag leaf	*A_Max_*	m^2^ leaf m^−2^ soil	0.005–0.01	≤40%	Fischer et al. (1998); Shearman et al. (2005)
Stay green	*S_G_*	–	0.00–1.50		
***Root water uptake***					
Rate of root water uptake	*R_u_*	%	1.0–5.0	Large variation	Asseng et al. (1998); Manschadi et al. (2006)
***Drought tolerance***					
Response of photosynthesis to water stress	*W_sa_*	–	0.1–1.0		
Maximum acceleration of leaf senescence due to water stress	*W_ss_*	–	1.0–1.7		

* Varietal difference in number of days till heading under long- and short-day conditions found between 9.74 and 107.40 in a photoperiodic response experiment (Kosner and Zurkova, 1996).

### 2.5 Target traits for improvement under the 2050-climate

A total of eight Sirius cultivar parameters related with different important plant traits, *viz*. growth rate, phenological development, response to abiotic stresses *etc*., were selected to design wheat ideotypes for increasing yield potential due to their a) large natural variations observed in wheat germplasms, b) potential for improvement through genetic adaptation, and c) importance in improving wheat yield under future climate change (Semenov et al., 2014; Semenov and Stratonovitch, 2013). The targeted traits are summarized in Table 3 and described briefly below.

#### 2.5.1 Phenology

The phyllochron (*P_h_*) is the thermal time required for the appearance of successive leaves, and day length response (*P_p_*) is the response of the final leaf number to day length (Brooking et al., 1995; Jamieson et al., 2007, 1998a, 1998b). An optimal flowering time and anthesis in relation to seasonal variations of temperature, solar radiation, day length and water availability are critical factors in maximizing grain yield (Akkaya et al., 2006; Richards, 2006). The rate of crop development and, consequently, the timing of anthesis and maturity could be altered by modifying *P_h_* and *P_p_* in plants (Jamieson et al., 2007; Semenov et al., 2014). The duration of grain filling (*G_f_*) is an important trait for increasing grain yield in wheat (Evans and Fischer, 1999). In Sirius, *G_f_* is defined as the cultivar-specific thermal time that needs to be accumulated to complete grain filling. During grain filling, assimilates for the grain are available from two sources, *viz*. new photosynthates produced from intercepted radiation and water-soluble carbohydrates stored mostly in the stem before anthesis. In Sirius, the labile carbohydrate pool is calculated as a fixed 25% of biomass at anthesis and is translocated to the grain during grain filling. Increasing *G_f_* will increase the amount of radiation intercepted by the crop and, consequently, grain yield. Under stress conditions, when grain growth could be terminated early as a result of leaves dying before the end of grain filling due to water or heat stress, grain yield will decrease not only because of the reduction in intercepted radiation, but also because not all of the labile carbohydrate pool will be translocated to the grain due to the shortage of time (Brooks et al., 2001; Semenov and Halford, 2009).

#### 2.5.2 Canopy

The rate of canopy expansion and achievement of the optimal canopy development and leaf area index are the key factors affecting the cumulative amount of intercepted radiation and transpiration demand during the growing season. The potential maximum area of flag leaf (*A_Max_*) is a key trait in modifying the rate of canopy expansion and the maximum achievable leaf area index (*LAI)*, which in turn will change the pattern of light interception and transpiration and, therefore, affect crop growth and final grain yield (Semenov and Stratonovitch, 2013; Semenov et al., 2014). On the other hand, reduced *A_Max_* could help to avoid drought stress by reducing transpiration and root water uptake. Delaying leaf senescence after anthesis is a possible strategy to increase grain yield by extending the duration of leaf senescence and maintaining the green leaf area longer: the so-called ‘stay green’ trait (*S_G_*) (Christopher et al., 2016; Luche et al., 2015; Silva et al., 2001).

#### 2.5.3 Root water uptake

In Sirius, we assume that only a proportion of available soil water can be extracted from each layer in the root zone by the plant on any day, depending on efficiency of water extraction (λ) and rate of root water uptake (*R_u_*). The proportion of daily water extractable by the plant declines from 10% at the top of the soil to *R_u_* at maximum root length. Faster root water uptake could reduce the current water stress experienced by plant in anticipation of additional available water later in the season in the form of precipitation or irrigation. In contrast, an alternative strategy of slower root water uptake might increase yield in dry environments by conserving water for successful completion of the life cycle (Manschadi et al., 2006).

#### 2.5.4 Drought tolerance

Water stress adversely affects both source and sink strength in plants. Photosynthesis and biomass production are reduced by water stress. New biomass production decreases proportionally to the response of photosynthesis to water stress (*W_sa_*). The rate of leaf senescence increases under water stress due to the modification in daily increment of thermal time by a factor termed maximum acceleration of leaf senescence due to water stress (*W_ss_*). Earlier leaf senescence will reduce grain yield due to reduction in intercepted radiation and photosynthesis and also reduction in translocation of the labile plant reserve carbohydrate to the grain due to premature termination of grain filling. Drought tolerance traits such as reduced *W_sa_* and *W_ss_* could be beneficial in rainfed conditions under drought (Semenov et al., 2009, 2014; Semenov and Halford, 2009).

### 2.6 Ideotype optimization under the 2050-climate

At each site in the UK and NZ, for both ideotypes, *IW_2050_* and *IP_2050_,* the eight selected parameters were optimized to maximize wheat yield under the 2050-climate (Table 3, Table 4). An evolutionary search algorithm with self-adaptation (EASA) was used in Sirius to optimize cultivar parameters in a high-dimensional parameter space with a complex fitness function for the best performance of crop ideotypes (Schwefel and Rudolph, 1995; Semenov and Terkel, 2003; Stratonovitch and Semenov, 2010). The EASA is a universal search optimization method, in which control parameters, inherited from a parent, determine variation in the target parameters, mutate randomly and independently, and evolve along with the target parameters to accelerate convergence (Semenov and Terkel, 2003). In each step of optimization, 16 new candidate ideotypes were generated from a ‘parent’ by perturbing its cultivar parameters randomly within the parameters’ ranges as defined in Table 3. As mentioned in “*Designing wheat ideotypes*”, the local winter wheat *cv*. *Claire* was used as a parent in our study, and the parameter ranges were based on model calibrations for existing modern wheat cultivars, allowing variations corresponding to the existing wheat germplasm (Table 3) (Akkaya et al., 2006; Kosner and Zurkova, 1996; Manschadi et al., 2006; Mosaad et al., 1995; Semenov and Halford, 2009; Shearman et al., 2005). Then for each candidate, yields were simulated for 100 years of future climate scenario. The candidate with the highest mean yield was selected as a parent for the next step. Candidates with a coefficient of variation (*CV*) of yield exceeding 10% or a harvest index (HI) over 0.64 were removed from the selection process. A *CV* of less than 10% guarantees high yield stability, which is a desirable trait in future cultivars, while the upper limit of HI was reported as 0.64 (Foulkes et al., 2011). The optimization process continued until no further improvement in yield potential was possible, or parameters converged to optimal values. To avoid convergence to a local maximum and to explore fully the parameter spaces, we initialized EASA with multiple ‘parents’. For each site, we used eight parents randomly scattered in the parameter space, except one parent that has the same cultivar parameters as *Claire*. For each of the eight initial parents, EASA converges to an optimal combination of parameters; the best of the eight was selected as an optimal ideotype for a selected site.

**Table 4:** Design of wheat ideotypes optimized for raising genetic yield potentials under 2050-climate (*HadGEM2*, *RCP8.5*) in water-limited (*IW_2050_*) and potential (*IP_2050_*) conditions in the United Kingdom (UK) and New Zealand (NZ).

Location	Country	Cultivar parameter†							
		*P_h_*	*P_p_*	*G_f_*	*A_Max_*	*S_G_*	*R_u_*	*W_sa_*	*W_ss_*
		(ºC day)	(Leaf h^−1^day length)	(ºC day)	(m^2^ leaf m^−2^ soil)	(-)	(%)	(-)	(-)
		Wheat ideotype designed under 2050-climate in water-limited condition (*IW_2050_*)							
Edinburgh	UK	120.0	0.0770	899.4	0.86 × 10^−2^	0.68	2.7	0.189	1.001
Leeds	UK	120.0	0.1310	900.0	0.71 × 10^−2^	1.00	3.2	0.100	1.338
Rothamsted	UK	119.9	0.0790	899.3	0.97 × 10^−2^	0.00	2.7	0.260	1.015
Gore	NZ	119.9	0.7240	779.1	0.72 × 10^−2^	0.31	3.6	0.756	1.226
Lincoln	NZ	120.0	0.7650	852.0	0.74 × 10^−2^	1.15	4.6	0.100	1.163
Pukekohe	NZ	120.0	0.8330	750.1	0.88 × 10^−2^	1.43	1.9	0.445	1.524
		Wheat ideotype designed under 2050-climate in potential condition (*IP_2050_*)							
Edinburgh	UK	120.0	0.0890	894.8	0.92 × 10^−2^	0.00	3.0	0.593	1.000
Leeds	UK	119.9	0.2230	874.4	1.00 × 10^−2^	1.07	4.3	0.100	1.700
Rothamsted	UK	120.0	0.0820	851.7	0.99 × 10^−2^	0.87	4.1	0.720	1.660
Gore	NZ	120.0	0.7420	873.8	0.74 × 10^−2^	0.79	3.3	0.726	1.382
Lincoln	NZ	120.0	0.7650	900.0	1.00 × 10^−2^	1.33	4.9	0.595	1.193
Pukekohe	NZ	120.0	0.8850	832.7	0.88 × 10^−2^	0.54	3.5	0.436	1.049

*P*_*h*_: Phyllochron.

*P*_*p*_: Day length response.

*G*_*f*_: Duration of grain filling.

*A*_*Max*_: Maximum area of flag leaf.

*S*_*G*_: Stay green.

*R*_*u*_: Rate of root water uptake.

*W*_*sa*_: Response of photosynthesis to water stress.

*W*_*ss*_: Maximum acceleration of leaf senescence.

† Other cultivar parameters remained the same as those of winter wheat *cv*. *Claire* (Table 2).

### 2.7 Simulation setup

We used Sirius version 2018, which is available from https://sites.google.com/view/sirius-wheat. A single soil-water profile, *Rothamsted,* with a total available water capacity of 210 mm, was used for all sites in the UK, and a single soil-water profile, *Lincoln,* with a total available water capacity of 270 mm, was used for all sites in NZ, to eliminate site-specific soil effects from the analysis. The soil profile was filled with the maximum available water capacity at sowing in both countries, the UK and NZ. Typical sowing dates of 20-October in the UK and 20-April in NZ were used. The performance of the current local winter wheat *cv*. *Claire*, as characterized before (Table 2), was simulated with water limitation under the baseline- (*CL_Base_*) and 2050-climate (*CL_2050_*) to provide a baseline for comparison and assess future climate impacts on *cv*. *Claire*. In Sirius, radiation use efficiency (*RUE*) is proportional to atmospheric [CO_2_], with an increase of 30% for doubling in [CO_2_] compared with the baseline of 338 ppm, which agrees with the recent meta-analysis of different field-scale experiments on the effects of [CO_2_] on crop (Vanuytrecht et al., 2012). A similar response is used by other wheat simulation models, such as CERES (Jamieson et al., 2000) and EPIC (Tubiello et al., 2000). For designing ideotype for the 2050-climate, a 10% increase in light use efficiency (*LUE*) was assumed (Zhu et al., 2010). Zhu et al. (2010) showed that 10% more carbon would be assimilated if the Rubisco specificity factor (λ) that represents the discrimination between CO_2_ and O_2_, is optimal under the current [CO_2_] level. Both wheat ideotypes, *IW_2050_* and *IP_2050_*, were optimized independently for each site for the maximum yield under 2050-climatic (*HadGEM2*, *RCP8.5*) condition. In all simulations, we assumed optimal agronomic managements, *e.g*. no N limitation or yield losses due to disease, pests or competition with weeds.

## 3 Results

### 3.1 Grain yield of cv. *Claire* under baseline- and 2050-climate

**Fig. 2:**
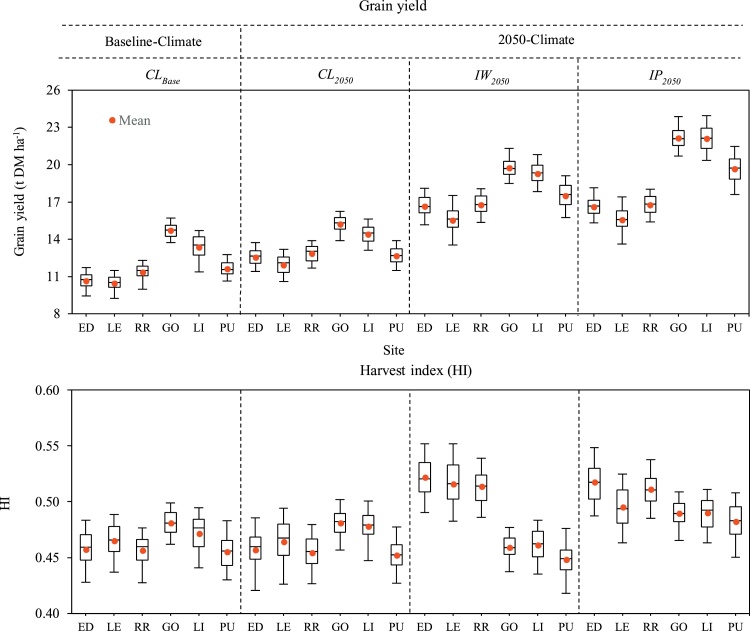
Simulated grain yield and harvest index (HI) of local winter wheat *cv*. *Claire* in the baseline- (*CL_Base_*) and 2050-climate (*CL_2050_*), and wheat ideotypes designed under 2050-climate in water-limited (*IW_2050_*) and potential (*IP_2050_*) conditions. The box plots show the 5-, 25-, 50-, 75- and 95-percentiles including mean. ED: Edinburgh (UK), LE: Leeds (UK), RR: Rothamsted (UK); GO: Gore (NZ), LI: Lincoln (NZ), PU: Pukekohe (NZ); UK: United Kingdom, NZ: New Zealand.

The averaged simulated grain yields of *Claire* in the baseline-climate (*CL_Base_*) were 10.8 and 13.2 t ha^−1^ in the UK and NZ respectively, indicating 22% (2.4 t ha^−1^) greater wheat yield in NZ than in the UK (Fig. 2). Yield variation (variance) among different sites was low (0.21 t ha^−1^) in the UK, whereas yield variation was greater (2.4 t ha^−1^) in NZ, with the highest yield at GO, followed by LI and PU (Fig. 2). Simulated yield of *Claire* under 2050-climate (*CL_2050_*) increased by 15% and 7% in the UK and NZ respectively, mainly due to CO_2_ fertilization and local climatic conditions. Grain yield of *CL_2050_* was 13% higher in NZ than in the UK.

### 3.2 Yield potential of the wheat ideotypes optimized under 2050-climate in water-limited and potential conditions

Table 4 shows the design of the wheat ideotypes for raising genetic yield potential of winter wheat in 2050-climate and Fig. 2 shows the simulated yield potentials of those ideotypes. The mean yield potentials of wheat ideotype *IW_2050_*, designed for the 2050-climate (*HadGEM2*, *RCP8.5*) under the water-limited condition, achieved 16.3 and 18.9 t ha^−1^ in the UK and NZ respectively. On average, yield potential of *IW_2050_* increased by 51% and 43% compared to *CL_Base_* and 31% and 34% than *CL_2050_* in the UK and NZ respectively. Grain yield potential of *IW_2050_* was 16% (2.6 t ha^−1^) higher in NZ than in the UK (Fig. 2). The 95-percentile *IW_2050_* yield in the UK was up to 18.1 t ha^−1^ at RR and ED, whereas the highest 95-percentile yield in NZ was 21.3 t ha^−1^ at GO. Like *CL_Base_*, site difference in yield potential (variance) of *IW_2050_* was small in the UK (0.49 t ha^−1^) but greater in NZ (1.4 t ha^−1^). The mean yield potential of wheat ideotypes *IP_2050_*, designed for the 2050-climate under the potential condition, increased by 51% and 62% than *CL_Base_* in the UK and NZ respectively. Additional mean yield benefit of *IP_2050_* over *IW_2050_* was minimum in the UK, but 13% in NZ. Overall, mean yield potential of *IP_2050_* was 31% (5 t ha^−1^) greater in NZ than in the UK.

### 3.3 Harvest index

The mean simulated HI for *Claire* under the baseline-climate ranged between 0.46 and 0.48 across the two countries, with almost no change in HI under the 2050-climate (Fig. 2). On average, a 13% increase in HI was found for *IW_2050_* (HI∼0.51-0.52) compared to *CL_Base_* in the UK, but decreased by 3% (HI∼0.45-0.46) in NZ. In contrast, HI increased by 11% and 4% in *IP_2050_* over *CL_Base_* in the UK and NZ respectively. HI of *IP_2050_* improved by 7% over *IW_2050_* in NZ.

### 3.4 Intercepted solar radiation

**Fig. 3:**
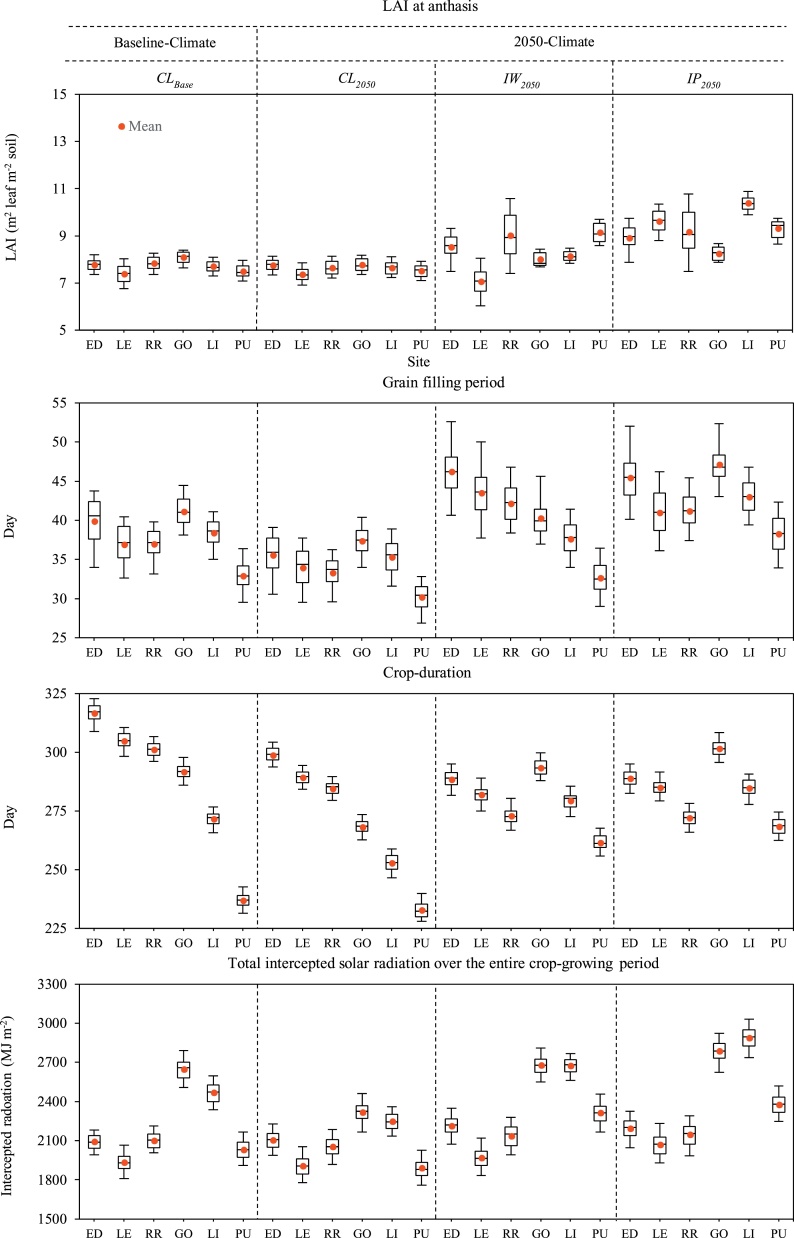
Simulated leaf area index (*LAI*) at anthesis, grain filling period, crop-duration and total intercepted solar radiation over the entire crop-growing period of local winter wheat *cv*. *Claire* in the baseline- (*CL_Base_*) and 2050-climate (*CL_2050_*), and wheat ideotypes designed under 2050-climate in water-limited (*IW_2050_*) and potential (*IP_2050_*) conditions. The box plots show the 5-, 25-, 50-, 75- and 95-percentiles including mean. ED: Edinburgh (UK), LE: Leeds (UK), RR: Rothamsted (UK); GO: Gore (NZ), LI: Lincoln (NZ), PU: Pukekohe (NZ); UK: United Kingdom, NZ: New Zealand.

The cumulative intercepted solar radiation over the entire wheat-growing period (sowing–maturity) of *Claire* decreased by 1% and 10% under 2050-climate compared to the baseline-climate in the UK and NZ respectively (Fig. 3). On contrary, intercepted radiation increased by 3–8% for *IW_2050_* and 5–13% for *IP_2050_*. Total intercepted radiations were 17%, 7%, 21% and 26% greater in NZ than in the UK for *CL_Base_*, *CL_2050_*, *IW_2050_* and *IP_2050_* respectively.

### 3.5 Crop canopy

Averaged simulated leaf area index (*LAI*) at anthesis of *CL_Base_* was 7.7 and 7.8 in the UK and NZ respectively (Fig. 3). Mean *LAI* remained almost the same for *CL_2050_* compared to *CL_Base_,* but increased by 7 and 9% for *IW_2050_* and 21% and 20% for *IP_2050_* in the UK and NZ respectively. On an average, *LAI* at anthesis was 1–3% greater in NZ than in the UK for *CL_Base_*, *CL_2050_*, *IW_2050_* and *IP_2050_*. Table 4 shows associated improvements in the optimized cultivar parameter ‘Maximum area of flag leaf’ *A_Max_* for *IW_2050_* and *IP_2050_*. On an average, 12% and 93% improvements were found in the ‘stay green’ trait *S_G_* for *IW_2050_* compared to *CL_Base_* in the UK and NZ respectively, whereas improvements in *S_G_* were 29% and 77% for *IP_2050_* (Table 4). The mean optimized *S_G_* value of *IW_2050_* was 72% greater in NZ than in the UK, whereas *S_G_* was 37% greater for *IP_2050_* in NZ than in the UK.

### 3.6 Wheat phenology

#### 3.6.1 Anthesis

The mean simulated anthesis date of *Claire* under baseline-climate was 26-June in the UK (250 DAS–days after sowing) and 15-November in NZ (209-DAS) (Table 5). Mean anthesis date of *Claire* was around 11-days earlier under 2050-climate in both countries. In contrary, mean anthesis date of *IW_2050_* was 31-days earlier than *CL_Base_* in the UK, but delayed by 15-days in NZ. Average anthesis date of *IP_2050_* delayed by about 2-days compared to *IW_2050_* in the UK, but remained almost the same in NZ. The mean anthesis date of *CL_Base_* was 41-days earlier in NZ than in the UK, but delayed by 5- and 3-days for *IW_2050_* and *IP_2050_* respectively. The optimized cultivar parameter Phyllochron *P_h_* increased to its maximum permissible value 120 °C day set in the optimization procedure for both the ideotypes in the UK and NZ (Table 4). In contrast, optimized mean day length response *P_p_* decreased by 74–81% in the UK, but increased by 55–60% in NZ for both the ideotypes.

**Table 5:** Sowing, anthesis and maturity dates of local winter wheat *cv. Claire* in the baseline- (*CL_Base_*) and 2050-climate (*CL_2050_*), and wheat ideotypes designed under 2050-climate in water-limited (*IW_2050_*) and potential (*IP_2050_*) conditions at study sites representing major wheat-growing regions across the United Kingdom (UK) and New Zealand (NZ).

Site^†^	Sowing	Anthesis		Maturity	
		Mean	SD (day)	Mean	SD (day)
*CL_Base_*					
ED	20 October	2 July	2.1	1 September	4.2
LE	20 October	25 June	2.3	21 August	3.6
RR	20 October	22 June	2.9	17 August	3.5
GO	20 April	4 December	2.3	5 February	3.5
LI	20 April	19 November	2.7	16 January	3.3
PU	20 April	21 October	3.1	13 December	3.6

*CL_2050_*					
ED	20 October	21 June	2.5	14 August	3.5
LE	20 October	14 June	2.6	5 August	3.2
RR	20 October	11 June	3.3	31 July	3.1
GO	20 April	16 November	2.3	13 January	3.4
LI	20 April	4 November	2.7	28 December	4.0
PU	20 April	22 October	3.8	8 December	4.0

*IW_2050_*					
ED	20 October	31 May	2.8	4 August	4.2
LE	20 October	28 May	2.8	29 July	4.5
RR	20 October	20 May	3.5	19 July	4.2
GO	20 April	9 December	2.2	7 February	3.6
LI	20 April	30 November	2.5	24 January	3.9
PU	20 April	18 November	2.3	6 January	3.6

*IP_2050_*					
ED	20 October	1 June	2.7	4 August	4.0
LE	20 October	3 June	2.6	1 August	3.6
RR	20 October	21 May	3.6	19 July	4.0
GO	20 April	10 December	2.1	15 February	3.9
LI	20 April	30 November	2.6	29 January	4.0
PU	20 April	20 November	2.0	13 January	3.9

Mean: Mean over 100 years.

SD: Standard deviation over 100 years.

†Note that the UK (United Kingdom) and NZ (New Zealand) are in the northern hemisphere and southern hemisphere respectively.

#### 3.6.2 Grain filling

Fig. 3 shows simulated grain filling duration of the current *cv. Claire* under baseline- and 2050-climate, and wheat ideotypes under 2050-climate in days, and Table 4 shows corresponding optimized cultivar parameter *G_f_* values in °C day for both the ideotypes. The mean simulated grain filling duration of *Claire* was 38-days under the baseline-climate in the UK and NZ, but reduced by 3-4-days under the 2050-climate (Fig. 3). The mean grain filling period of *IW_2050_* extended by 6-days compared to *CL_Base_* in the UK, but remained the same in NZ. Averaged grain filling duration of *IP_2050_* increased by 5-days compared to *CL_Base_* in both UK and NZ. There was almost no difference in average grain filling duration between the countries for *CL_Base_*, *CL_2050_* and *IP_2050_*, except *IW_2050_* for which grain filling duration was 7-days shorter in NZ than the UK.

#### 3.6.3 Maturity

Averaged maturity dates of *Claire* under baseline-climate were 23-August (308-DAS) and 11-January (267-DAS) in the UK and NZ respectively (Table 5). Mean total wheat-growing period (sowing∼maturity) of *Claire* shrank by 5–6% (16-17-days) under 2050-climate in both countries compared to *CL_Base_*. On average, total wheat growing period of *IW_2050_* extended by 5% (11-days) over *CL_Base_* in NZ, but shortened by 9% (27-days) in the UK. Similarly, wheat growing-period of *IP_2050_* increased by 7% (∼18-days) over *CL_Base_* in NZ, but shortened by 8% (26-days) in the UK. Overall, mean wheat growing-period of *Claire* was 13–14% (40-41-days) shorter in NZ than in the UK under the baseline- and 2050-climate. Whereas, the difference in growing-period between the countries decrease to 1% (3-days) for both the ideotypes, but mean growing-period was 3-days longer for *IP_2050_* in NZ than the UK, whereas 3-days shorter for *IW_2050_* in NZ than the UK.

### 3.7 Root water uptake and drought tolerance traits

The mean optimized ‘rate of root water uptake’ *R_u_* in *IW_2050_* reduced by 4% compared to *CL_Base_* in the UK, but increased by 12% in NZ (Table 4). On the other hand, *R_u_* in *IP_2050_* increased by 27% and 30% in the UK and NZ respectively. Averaged *R_u_* was 3–17% higher in NZ than in the UK. The changes in the drought tolerance traits, ‘response of photosynthesis to water stress’ *W_sa_* and ‘maximum acceleration of leaf senescence due to water stress’ *W_ss_*, were not important both in the UK and NZ as winter wheat in the present study sties hardly faced any critical drought stress under 2050-climate (Table 4).

## 4 Discussion

### 4.1 Simulated grain yield of cv. *Claire* under baseline and future climate

The mean simulated grain yields of the local winter wheat *cv*. *Claire*, used in our study, were 10.8 t ha^−1^ in the UK and 13.2 t ha^-1^ in NZ under the baseline (1980–2010) climatic condition, which is 2.6–3.4 times greater than the global average wheat yield of 3 t ha^-1^ (FAOSTAT, 2018). High winter wheat yield in the UK and NZ could be linked to low air temperature (9–15 °C) and sufficient precipitation (596–1296 mm yr^-1^), with very few or almost no extreme climatic events and abiotic stresses (e.g., heat and drought stresses), which ensure slow growth and longer crop maturity (237–317 days). These results in greater cumulative intercepted solar radiation over the growing season, and higher total crop biomass and grain yields in these two high-latitude (56 °N–46 °S) countries. For similar reasons, the highest wheat productive countries are generally lie at high latitudes, for examples, Ireland, Belgium, the Netherlands, including NZ and the UK (FAOSTAT, 2018; Hawkesford et al., 2013). The mean simulated wheat yields in our study are 39–57% greater than the national averages of the UK and NZ (FAOSTAT, 2018). This could be explained by our assumptions about agronomic management practices that are effective in meeting the N demand and achieving full control of any weed, disease and pest infestations, factors that may reduce the national mean wheat yield. The mean baseline wheat yields in the present study are close to good year wheat yields (8–14 t ha^-1^) as reported by various studies across the UK and NZ (Carmo-Silva et al., 2017; Craigie et al., 2015; Curtin et al., 2008; Perryman et al., 2018; Roques et al., 2017). Different studies reported similar yield potentials of current wheat cultivars under optimal managements in high productive countries at high latitude (Boogaard et al., 2013; Schils et al., 2018).

Yield of cereal crops including wheat is generally predicted to reduce under future climate change due to mainly increase temperature, changing precipitation pattern and quantity, and increasing climate extreme events, which are supposed to erode positive impacts of CO_2_ fertilization under future climatic condition (Asseng et al., 2015; Liu et al., 2016). Our simulation study predicts that anthesis of *Claire* will be early and total crop growing period as well as grain filling duration will be shortened under 2050-climate both in the UK and NZ, resulting reduction in cumulative intercepted solar radiation (1–10%). These can be explained by an increase in air temperature which speeds up the crop phenological development. Reduction in total intercepted radiation reduces total photosynthesis, biomass production and ultimately grain yield. Nevertheless, grain yield of *Claire* increased by 7–15% under 2050-climate in our study, as CO_2_ fertilization ([CO_2_] 541 ppm, 49% increase compared to baseline [CO_2_] of 364 ppm) overrides the negative impacts of reduced intercepted radiation in the UK and NZ. Many studies reported an increase in wheat production due to CO_2_ fertilization under future climatic conditions in high productive countries at high latitude, such as NW-Europe including the UK (Semenov and Shewry, 2011; Webber et al., 2018).

### 4.2 Wheat ideotypes designed for raising genetic yield potential under 2050-climate

In the present study, wheat ideotypes were optimized for raising genetic yield potential under 2050-climate (*HadGEM2*, *RCP8.5*) in both water-limited (*IW_2050_*) and potential conditions (*IP_2050_*) in the two high productive countries *viz*. the UK and NZ. Modelling predicts the possibility of increasing mean yield potential of winter wheat to 16-19 t ha^−1^ (43–51%) under 2050-climate in water limited condition in the UK and NZ, whereas 16-21 t ha^-1^ (51–62%) in the potential condition. Various experimental and review studies indicated necessity of crop improvement, genetic adaptation and new cultivars for increasing the yield potential of wheat under climate change (Reynolds et al., 2011; Semenov and Halford, 2009; Semenov et al., 2014). Mitchell and Sheehy (2018) have recently indicated that potential wheat yield could reach to 20 t ha^-1^ in the UK under optimal condition from new wheat cultivars. Craigie et al. (2015) obtained wheat yield a maximum of 15.9 t ha^-1^ in their experimental fields in NZ and believe that further increase is possible with the improved future cultivars.

The increase in yield potential of both the wheat ideotypes (*IW_2050_* and *IP_2050_*) compared to *Claire* is due to the improvements in both the sources (e.g., photosynthate and biomass) and sink sizes (e.g., grain number) resulting from optimised cultivar parameters and plant traits under 2050-climate. The optimized canopy structure under 2050-climate, in terms of *LAI* at flowering and ‘stay green’ trait *S_G_*, is one reason of increasing genetic yield potential of both ideotypes compared to present *cv. Claire*. Greater *LAI* leads to greater intercepted radiation and photosynthesis, whereas improved *S_G_* is an important trait for delaying leaf secession, maintaining the green leaf tissue longer after anthesis for photosynthesis, and increasing biomass, HI and grain yield under both water-limited and potential conditions (Christopher et al., 2016; Foulkes et al., 2007; Luche et al., 2015; Richards, 2006; Semenov and Stratonovitch, 2013). Improved grain filling duration is another important trait for raising yield potentials of both ideotypes. Extending grain filling period not only increases intercepted radiation and photosynthesis after anthesis for direct grain filling, but also increase the chance of complete translocation of plant-reserved labile carbohydrate into grain, and thus improved HI and grain yield (Brooks et al., 2001; Semenov and Stratonovitch, 2013). The phyllochron *P_h_* and day length response *P_p_* are two plant traits which mostly control the rate of crop phenological development, and the timing of anthesis and maturity (Brooking et al., Jamieson et al., 1998a). The rate of crop development, and anthesis and maturity time could be manipulated by modifying *P_h_* and *P_p_* (Jamieson et al., 2007; Semenov et al., 2014). Increased *P_h_* in both the ideotypes, *IW_2050_* and *IP_2050_*, is beneficial for keeping the rate of crop development lower under higher temperatures in the 2050-climate. A slower phenological development supports a longer crop duration, resulting in greater intercepted radiation and crop yield. An optimal anthesis date is one of the key traits for high yield potential, as favourable environmental conditions at anthesis promotes greater primary grain seating (Hills and Li, 2016). Timing of athesis (early/late) influences also biomass production and labile carbohydrate reserve at anthesis. Our results show that a late anthesis is beneficial for high yield potential of both ideotypes under 2050-climate in NZ, but an early anthesis is important in the UK. Additional model runs with the late anthesis under 2050-climate in the UK revealed that a higher temperature during grain filling was the main reason for compromise between early anthesis and extended grain filling duration for a better yield potential in the UK, whereas no such limitation was found in NZ. Present results predict that longer wheat growing season compared to *Claire* is important under 2050-climate for high yield potential of both ideotypes particularly in NZ. The delayed anthesis and maturity help to increase total intercepted solar radiation, photosynthesis, biomass production and grain yield NZ (Hawkesford et al., 2013; Semenov and Stratonovitch, 2013). However, a shorter wheat-growing season than the current *cv. Claire* is beneficial to optimize anthesis, grain filling duration, intercepted radiation and ultimately the grain yield under 2050-climate in the UK. Relatively longer day length during wheat growing period in the UK than NZ due to the higher latitude is another constraint in increasing leaf number, and hence delaying anthesis and extending maturity in the UK. In Sirius, final leaf number before anthesis decreases with increasing day length, resulting in a shorter vegetative stage and early flowering and maturity (Brooking et al., 1995; Jamieson et al., 1998b). Increased optimized trait ‘rate of root water uptake’ *R_u_* in both the ideotypes in NZ and *IP_2050_* in the UK compared to *Claire* shows that improvement in rate of root water uptake is helpful to increase biomass and grain yield potentials and to satisfy any water stress particularly under potential condition in both countries. On the contrary, a reduced rate of root water uptake would be an important strategy for successful completion of the crop life-cycle along with best possible yield under 2050-climate in water-limited condition particularly in the UK (Manschadi et al., 2006; Senapati et al., 2018). However, no such limitation was found under water-limited condition in NZ. Other reasons for the greater yield potentials of both ideotypes in the 2050-climate compared to present yield of *Claire* are the CO_2_ fertilization effect and *LUE* improvement, as mentioned in the section ‘Simulation setup’. It is worth mentioning that HI was not optimized directly in our study. The mean HI of both ideotypes ranged between 0.45 and 0.52, whereas the upper theoretical limit of HI reported as 0.64 (Foulkes et al., 2011). Thus, our results indicate that there is still an additional possibility to raise genetic yield potential of wheat further by optimising partitioning of assimilate to grain while maintaining lodging resistance.

A 13% additional yield potential was obtained for winter wheat under 2050-climate in potential condition compared to water-limited condition in NZ, whereas almost no such yield benefit was achieved in the UK. These results indicate that wheat ideotype designed for the rainfed condition in the UK has the best possible yield potential under the future climatic condition, and it is not possible to push the yield potential up further by designing a separate ideotype for the non-water-limited condition due to the local climatic limitations. On the other hand, it is possible to achieve greater yield potential of wheat in NZ by designing wheat ideotypes separately for water-limited and optimal conditions due to the local climatic advantages. A higher yield potential of wheat under optimal than water-limited condition particularly in NZ could be explained by relatively better optimized cultivar parameters and crop-physiology (Table 4, Fig. 3).

Although optimized grain filling duration and total wheat growing-period were nearly the same in both countries for both ideotypes, better canopy structure (e.g., *LAI* and *S_G_*), greater root water uptake and a bit delayed anthesis provides extra benefits for greater intercepted radiation, biomass production and grain yield potentials in NZ than in the UK (Table 4, Fig. 3, Supplementary Fig. S1). The greater *LAI* at anthesis in NZ is mainly due to the greater number of leaves, which is associated with shorter day length in NZ due to its lower latitude (Brooking et al., 1995; Jamieson et al., 1998b). However, the main reason of greater genetic yield potentials of both ideotypes in NZ compared to the UK could be explained by higher cumulative intercepted radiation over the wheat-growing period (Fig. 3) due to greater mean annual solar radiation (40–48%) across our study sites in NZ (southern hemisphere, 37–46 °S) than the UK (northern hemisphere, 52–56 °N). Greater mean annual solar radiation in NZ compared to the UK could be explained by lower latitude (≤10°), thinner O_3_ layer and lower pollution in NZ, and the asymmetric elliptical shape of the earth’s orbit, which brings the southern hemisphere closer to the sun during the southern summer than the northern hemisphere during the northern summer (NASA, 2009). A positive relationship has been reported between solar radiation and wheat yield, whereas a negative relationship has been found between wheat yield and air pollution (Ahmed and Fayyaz-ul, 2011; Chen et al., 2013; Gupta et al., 2017). Greater crop biomass resulting from higher intercepted radiation has been reported and reviewed by various researchers (Hawkesford et al., 2013; Jamieson et al., 1998b; Reynolds et al., 2009). Greater plant biomass in NZ (Supplementary Fig. S1) increased the availability of assimilates for ear formation, resulting in greater spikelet and grain numbers and ultimately higher wheat yields than in the UK (Hawkesford et al., 2013; Reynolds et al., 2009). Another reason for greater biomass and grain yield in NZ than in the UK could be a higher photothermal quotient (solar radiation/air temperature) in NZ (Supplementary Fig. S2). Although averaged annual air temperature was 2.4–2.7 °C greater in NZ than in the UK, the photothermal quotient was 18% greater in NZ than in the UK. Total crop biomass, as well as grain yield, was found to increase with an increasing photothermal quotient (Brooks et al., 2001; Nalley et al., 2009; Wolf et al., 1996). Thus, local climatic advantages, such as greater solar radiation and photothermal quotient, and shorter day length, are the main drivers of greater wheat yield potential in NZ, whereas lower solar radiation and photothermal quotient and longer day length are the constraints in raising yield potential in the UK to the same extent as in NZ. Different local climatic advantages and limitations on wheat yield had been found at various locations around the world, for examples, solar radiation, photothermal quotient, day length, high temperature etc. (Ahmed and Fayyaz-ul, 2011; Brooking et al., 1995; Chen et al., 2013; Lollato et al., 2017; Nalley et al., 2009). The greater grain yield potentials of winter wheat in NZ than in the UK has been reported by various studies (Craigie et al., 2015; Curtin et al., 2008; Defra, 2017; FAOSTAT, 2018).

### 4.3 General discussion

Designing crop ideotypes, based on crop physiological knowledge, has recently been reviewed and prioritized in order to raise the genetic yield potential of wheat and other cereals under climate change (Foulkes et al., 2011; Hawkesford et al., 2013; Parry et al., 2011; Reynolds et al., 2011, 2009). We have identified the important cultivar parameters and plant traits ranging from phenology, canopy structure to root water uptake, and calculated their optimal combinations quantitively to achieve the best possible wheat yield under targeted climatic condition in 2050 (*HadGEM2*, *RCP8.5*) in the UK and NZ. We have optimized wheat ideotypes by using the full parameter ranges in a multidimensional space of cultivar parameters and plant traits, considering the basis of crop physiology and the existence of natural genetic variations in wheat germplasm (Table 3). In our simulation experiments, we assumed that cultivar parameters selected for optimization (Table 3) could be changed independently from each other by the EASA evolutionary algorithm during optimization process. This might not be always the case. For example, a high value for maximum area of flag leaf *A_max_* may require a higher value for phyllochron *P_h_,* to provide sufficient time for larger leaves to grow. Dependencies between parameters or any other constrains, if known, can be incorporated in the current modelling framework in the same way as we accounted for restrictions in the maximum value of harvest index HI or yield coefficient of variation *CV*. Nevertheless, once parameters sampled from the parameter spaces, all known interactions and trade-offs among them were taken into account within the Sirius model. Some of the cultivar parameters, such as phyllochron *P_h_,* which determined phenological development and strongly affected grain yield, were subject to strong selection pressure and converged to an optimal single value for all sites. Some other parameters, such as ‘stay green’ *S_G_* or ‘response of photosynthesis to water stress’ *W_sa_*, did not converged to an optimal value at individual site, because their values had smaller effects on grain yield. Rather, their evolution represented a “random walk”. As a result, their final values varied substantially between sites (Table 4). Ranges of parameter values for optimization, where possible, were determined from existing genetic variations reported in the literature (Table 3). However, for some parameters, such as drought tolerance, *W_sa_* and *W_ss_*, such information was not available. In this case, we defined range of parameter values by using cultivars previously calibrated for Sirius in diverse environments. We designed wheat ideotypes for 2050-climate using climate projection from a single global climate model (GCM), *HadGEM2*, and a single emission scenario, *RCP8.5*. Design of ideotype may change if a different GCM or RCP is selected from the CMIP5 ensemble. To assess uncertainty in ideotype design related to uncertainty in future climate, ideally design should be run for the entire CMIP5 ensemble due to nonlinear nature of the Sirius wheat model. However, CMIP5 includes over 50 variants of GCMs, which sample uncertainties in model structures and initial conditions, and four emission scenarios, *RCP2.6*, *RCP4.5*, *RCP6.0* and *RCP8.5*. For computationally demanding tasks, such as ideotype design, it is not practical to explore all possible combinations of GCM and RCP. A potential solution to reduce number of simulations could be the use of climate sensitivity index to select a subset of GCMs which preserves the range of uncertainty found in CMIP5 itself (Semenov and Stratonovitch, 2015). This would allow to quantify uncertainty in design resulting from the CMIP5 ensemble by conducting fewer simulation experiments. Nevertheless, the design of wheat ideotypes in our study could be used as a road map by plant breeders for future wheat improvement and genetic adaptation to increase yield potential under future climatic conditions in the UK and NZ. Recent advances in annotated reference wheat genome (IWGSC, 2018), modern plant breeding technologies (e.g., wide crossing and resynthesis, molecular-marker-assisted breeding, QTL-mapping, genomics-assisted breeding, chemical and genetic modulation, gene-editing, etc.) (Breseghello and Coelho, 2013; Kole et al., 2015; Rauf et al., 2015; Reynolds et al., 2009) and the existence of large natural genetic variation in the target traits of wheat (Tables 3,4) could help plant breeders to breed desirable wheat cultivars based on ideotypes optimized for the target future climate. Constraints or difficulties in improving certain traits and achieving any given combinations physiologically or genetically could be incorporated in the optimization procedure by reducing the range for such parameters, or introducing dependencies between parameters.

In conclusion, modelling predicts the possibility of raising genetic yield potential of winter wheat by 43–51% in 2050-climate (*HadGEM2*, *RCP8.5*) under water limited condition in the UK and NZ, whereas under potential condition, up to 51–62% increase could be achieved. NZ would have higher yield potential (16–31%) under both the water-limited and potential conditions due to the local climatic advantages compared to the UK, but wheat ideotypes need to be designed separately for NZ to make greatest use of the local climatic conditions. Although recorded wheat yields in the UK and NZ are one of the highest, our study demonstrates the possibility of substantially increasing the genetic wheat yield potential under future climatic conditions. The design of wheat ideotypes in the present study provides plant scientists and wheat breeders with a possible road map for selection of the target traits and their optimal combinations quantitatively for wheat improvement and genetic adaptation to increase the yield potential of wheat under climate change. The method of designing wheat ideotypes to raise yield potential under future climatic conditions in our study is generic in nature, and therefore it could be applicable globally. However, the extent of possible yield improvement would depend on local climatic and environmental conditions.
