# Supplementary material for: Raising genetic yield potential in high productive countries: Designing wheat ideotypes under climate change
Source: Agric For Meteorol. 2019 Jun 15;271:33–45. doi: 10.1016/j.agrformet.2019.02.025 (PMC6559216; doi:10.1016/j.agrformet.2019.02.025)
Supplement: Supplementary file 1 [file mmc1.docx]

**Appendix A. Supplementary data**


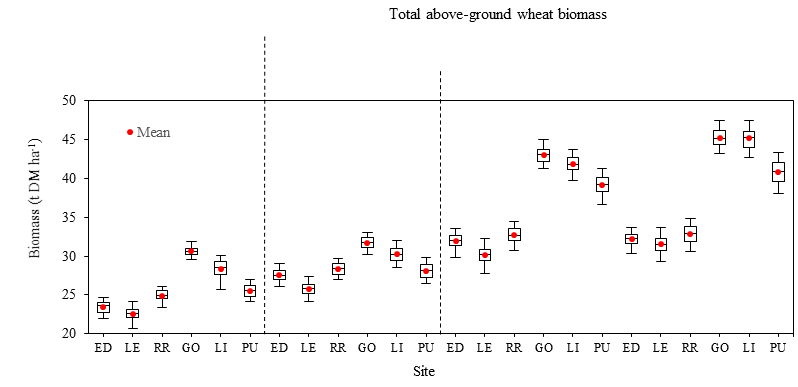


*CL_Base_*

*CL_2050_*

Baseline-Climate

2050-Climate

*IW_2050_*

*IP_2050_*

**Fig. S1.** Total aboveground crop biomass of local winter wheat *cv*. *Claire* under baseline- (*CL_Base_*) and 2050-climate (*CL_2050_*), and wheat ideotypes designed under 2050-climate in water-limited (*IW_2050_*) and potential (*IP_2050_*) conditions. The box plots show the 5-, 25-, 50-, 75- and 95-percentiles including mean. ED: Edinburgh (UK), LE: Leeds (UK), RR: Rothamsted (UK); GO: Gore (NZ), LI: Lincoln (NZ), PU: Pukekohe (NZ); UK: United Kingdom, NZ: New Zealand.

**Fig. S2.** Photothermal quotient in the United Kingdom (UK) and New Zealand (NZ) in the baseline-climate, and the 2050-climate. Note that the UK (northern hemisphere) and NZ (southern hemisphere) are in opposite hemispheres.
